# Making Sense of Mobile Health Data: An Open Architecture to Improve Individual- and Population-Level Health

**DOI:** 10.2196/jmir.2152

**Published:** 2012-08-09

**Authors:** Connie Chen, David Haddad, Joshua Selsky, Julia E Hoffman, Richard L Kravitz, Deborah E Estrin, Ida Sim

**Affiliations:** ^1^School of MedicineUniversity of California San FranciscoSan Francisco, CAUnited States; ^2^Open mHealthSan Francisco, CAUnited States; ^3^Department of Computer ScienceUniversity of California Los AngelesLos Angeles, CAUnited States; ^4^National Center for Post-Traumatic Stress DisorderVA Palo Alto Healthcare SystemMenlo Park, CAUnited States; ^5^Division of General Medicine and Center for Healthcare Policy and ResearchUniversity of California DavisSacramento, CAUnited States; ^6^Division of General Internal MedicineDepartment of MedicineUniversity of California San FranciscoSan Francisco, CAUnited States

**Keywords:** Mobile health, software tools, software engineering, open access to information, open architecture, open source, evaluation methodology, data analysis, data visualization

## Abstract

Mobile phones and devices, with their constant presence, data connectivity, and multiple intrinsic sensors, can support around-the-clock chronic disease prevention and management that is integrated with daily life. These mobile health (mHealth) devices can produce tremendous amounts of location-rich, real-time, high-frequency data. Unfortunately, these data are often full of bias, noise, variability, and gaps. Robust tools and techniques have not yet been developed to make mHealth data more meaningful to patients and clinicians. To be most useful, health data should be sharable across multiple mHealth applications and connected to electronic health records. The lack of data sharing and dearth of tools and techniques for making sense of health data are critical bottlenecks limiting the impact of mHealth to improve health outcomes. We describe Open mHealth, a nonprofit organization that is building an open software architecture to address these data sharing and “sense-making” bottlenecks. Our architecture consists of open source software modules with well-defined interfaces using a minimal set of common metadata. An initial set of modules, called InfoVis, has been developed for data analysis and visualization. A second set of modules, our Personal Evidence Architecture, will support scientific inferences from mHealth data. These Personal Evidence Architecture modules will include standardized, validated clinical measures to support novel evaluation methods, such as n-of-1 studies. All of Open mHealth’s modules are designed to be reusable across multiple applications, disease conditions, and user populations to maximize impact and flexibility. We are also building an open community of developers and health innovators, modeled after the open approach taken in the initial growth of the Internet, to foster meaningful cross-disciplinary collaboration around new tools and techniques. An open mHealth community and architecture will catalyze increased mHealth efficiency, effectiveness, and innovation.

## Introduction

Today, mobile phones are in nearly every pocket, with an estimated 83% of Americans owning a mobile phone and 35% of US mobile phone subscribers possessing a smartphone [1]. Mobile phones and devices—with their constant presence, connectivity, and multiple intrinsic sensors—can be easily integrated into daily life to effectively support chronic disease prevention and management.

Early research suggests that mobile health (mHealth) applications can empower individuals to track and manage their own health, thus improving user-centered outcomes. A recent randomized controlled trial of WellDoc, a diabetes management app that prompts users via short message service text messages to check and record their blood sugar values, showed a significant reduction in glycated hemoglobin among users at 1 year (1.9% in the treatment group versus 0.7% for usual care, *P *< .001), as well as a 20% reduction in emergency department use and hospitalization [2,3]. Text message reminders have also been shown to promote smoking cessation, improve attendance at medical appointments, increase knowledge about prenatal care, and encourage sunscreen use. Patients are increasingly using mobile phones to track their own health measures, ranging from blood sugar to vital signs to exercise and food intake [4-10].

Despite the promise of these preliminary findings, the evidence base for mHealth remains sparse and methodologically weak [11]. Anecdotally, the rates of reuse for mobile applications remain very low [12]. With nearly 12,000 health-related apps available, and more being created every day, the continued proliferation of mHealth apps runs the risk of simply creating confusion [13]. It is predicted that the number of mobile app downloads will reach 142 million by 2016, generating billions of real-world data points on patient health experiences and outcomes [14].

Unfortunately, the mHealth ecosystem lacks modular tools and techniques for drawing meaning and scientifically valid inferences from the masses of collected data. Without the development of more sophisticated and effective tools for data visualization and analysis, legitimate questions remain regarding mHealth’s projected impact on chronic disease management and prevention. In considering how the mHealth ecosystem might need to evolve to achieve maximum impact, we can draw lessons from the success of the Internet’s open architecture and its ability to support both open and closed proprietary applications. In contrast, the closed, stovepipe architecture of electronic health records yields a cautionary tale about the deleterious effects of highly closed ecosystems.

In this paper, we describe Open mHealth, a nonprofit organization that is building an open software architecture for mHealth and catalyzing an open community of developers, clinicians, researchers, and entrepreneurs to build and reuse Open mHealth modules across a broad range of mHealth applications, disease conditions, and user populations. Over time, the open architecture’s functionality and robustness will grow through reuse and community validation. Our postulate is that progress in mHealth will be best served by a dynamic, open, multidisciplinary community that innovates collaboratively on an open architecture.

## How mHealth Data Contribute to Three Essential Feedback Loops

mHealth applications are rich sources of passive and actively collected data. These mHealth data are integral to three essential feedback loops for improving health outcomes: (1) patient-facing feedback to guide patients’ self-care (eg, how does taking the medication topiramate affect my pain?), (2) clinician-directed summary data to guide clinical decision making for individual patients (eg, how do the side effects and therapeutic benefits of topiramate balance for my patient?), and (3) research evidence to improve clinical care for groups of patients (populations) (eg, in patients with neuropathic pain, does topiramate reduce pain intensity and improve quality of life?).

These three feedback loops are powerful channels by which mHealth data can improve health outcomes. However, mHealth data tend to have lots of bias, noise, variability, and gaps, such that it is difficult to make sense of the data and extract relevant features and patterns to drive information through the feedback loops. Lack of visualization tools to help end users understand collected data and absence of analysis tools for generating robust clinical evidence remain significant impediments threatening to limit the impact of mobile technology on health outcomes.

In addition, the disaggregation of data across siloed applications and devices hinders patient-specific analysis. For example, a diabetic patient might find herself recording her insulin use, nutrition intake, exercise, blood sugar values, and mood in five separate mHealth applications. Without a shared architecture for data analysis, the patient and clinician would encounter significant friction in trying to correlate the blood sugar values with corresponding diet, exercise, or medication data. Without being able to determine what was driving a suboptimal blood sugar value, the clinician would not be able to make a fully informed adjustment to the patient’s management plan and might eventually discourage her patients from sharing this type of uninterpretable data.

On top of data aggregation and analysis, there must be visual displays that help users—both patients and providers—understand the meaning of their data. The lack of heterogeneous-data analysis tools among mHealth applications is similarly limiting the use of these data for clinical research [15]. This limitation presents a very high opportunity cost. Unlike traditional randomized controlled studies, which are costly, slow, and generate estimates of average treatment effects, trials of mHealth applications can be conducted as time series and n-of-1 studies for individual patients, enabling researchers to estimate with a high degree of granularity within-individual correlations between clinical interventions, specific patient behaviors, and health outcomes [16].

## Open mHealth: An Open Architecture to Improve Individual- and Population-Level Health Outcomes

Open mHealth addresses the gap between the reality of fragmented mHealth applications and the full promise of mHealth powering the three feedback loops of personal care, clinical decision making, and research evidence in a virtuous cycle. Features of the needed solution include the following:

- Community: must be multidisciplinary, safe, and collaborative
- Iteration: delivers efficient reuse through collaborative cycles of development
- Flexible architecture: recognizes both the limits and the utility of existing closed systems and is designed to maximize participation from all players
- Shared learning: uses the strongest appropriate methods, matched to the evidence needs and the rapid pace of technological advances in mHealth
- Scalable solutions: offers mass customization of applications and evidence, from personal to population.

**Figure 1:**
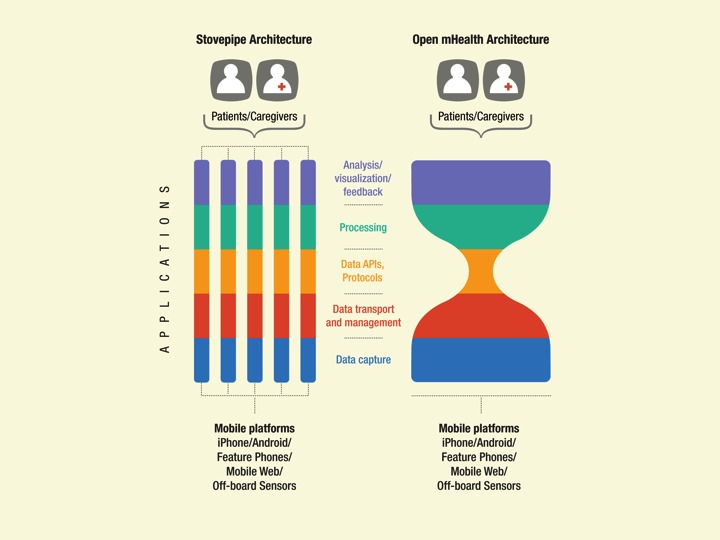
Stovepipe versus open architectures: mHealth apps (left) are built independently with little sharing of data, methods, or learning. In contrast, the Internet has an hourglass architecture (right), in which a common protocol, transmission control protocol/Internet protocol (TCP/IP), acts as a simple point of commonality at the narrow waist that allows innovation to flourish through open application programming interfaces (APIs) both above and below the waist. Open mHealth aims to catalyze the mHealth ecosystem from a siloed architecture to an hourglass architecture to increase the scale and effectiveness of mHealth.

As is the case for the Internet, shared modules with open application programming interfaces (APIs) around a minimal set of common standards meet these needs for an open community. The Internet has what is called an hourglass architecture, from which it derived much of its success. In this architecture, a common communications protocol acts as a simple point of commonality at the narrow waist. This allows innovation to flourish through open interfaces, or APIs, both above and below the waist (Figure 1).

More recent examples of successful open software communities are Apache, Eclipse, and Mozilla. These communities spawned huge, lucrative industries through collaborative development that blended both proprietary and open components. mHealth is ripe for such open treatment.

## InfoVis

**Figure 2:**
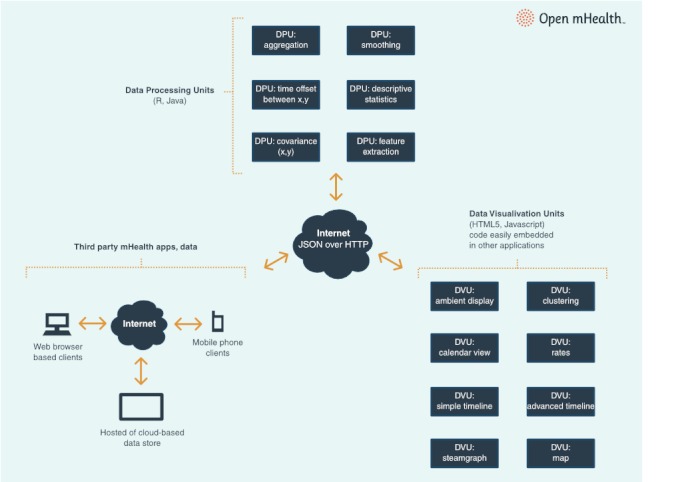
InfoVis: Third-party data applications and data stores save and manage data. Data processing units (DPUs) are the building blocks for extracting relevant features and patterns from data streams. Data visualization units enable visual presentations to be created from the extracted data features. Each DPU and DVU completes one task, and then can be composed for higher functions. HTTP = hypertext transfer protocol, JSON = JavaScript object notation.

Open mHealth is catalyzing a decentralized, innovative community committed to developing sharable mHealth tools with open APIs that allow independently developed software components to be mixed and matched, swapped, and shared like Lego blocks (Figure 2) [17]. To begin, we developed InfoVis, which is the architectural scaffolding for data analysis and visualization building blocks that the Open mHealth community is creating, combining, evaluating, and adapting.

Open mHealth’s architecture mimics the natural structure of the honeycomb. The foundational framework is a common set of principles and APIs that enable reusable software modules—or individual pieces of the honeycomb—to be built into and upon the underlying structure in a plug-and-play fashion. The architecture enables additional modules to be easily added and pieced together, facilitating the growth of the entire honeycomb and strengthening the overall structure.

The basic types of reusable software components in Open mHealth are data processing units (DPUs) and data visualization units (DVUs). DPUs are the building blocks for extracting relevant features from data streams, whereas DVUs enable the presentation of those features and patterns. Data storage units are components that manage the input and output of data to DPUs and DVUs, and are specific to particular data storage solutions (eg, a Health Insurance Portability and Accountability Act [HIPAA]-compliant cloud storage vendor). For any particular application, the DPUs, DVUs, and data storage units are embedded in a plug-and-play fashion within that application’s running system, which can range from the Android operating system, for example, to full-featured platforms such as that of AT&T [18].

Each DPU and DVU does one task and can be composed to produce higher-level functions. For example, low-level DPUs can transform time series of on-phone and other sensor measurements (eg, accelerometer) into time series of user states (eg, sitting, walking, or driving). Midlevel DPUs compute clinically relevant metrics (eg, 6-minute walk test) [19]. Higher-level DPUs process and fuse one or more metrics (eg, activity metrics with self-report data) to come up with health markers for a person’s state (eg, functional status). Such hierarchical analyses that transform lower-level data streams into higher-order markers will reduce the need for self-reports, thus mitigating the challenge of user engagement.

Open mHealth components can be incorporated into applications as libraries or can be invoked using JavaScript object notation over hypertext transfer protocol if they are developed with a Web service wrapper. We encourage component developers to support both library- and Web services-based approaches to accommodate application-specific preferences. All components must follow interoperability specifications that set forth common patterns of implementation and methods for data interchange. These specifications, which are continually being refined and are available at [20], follow these principles: using modern open source industry standards where possible; using lightweight interoperability standards; using declarative semantics with allowance for multiple bindings to multiple reference standards; and allowing standards to emerge through community patterns of use rather than imposition. For example, the data input to a DVU is at a minimum specified by a payload ID in the Open mHealth namespace (omh). This payload ID will be in the form of a string (eg, omh:serum-sodium) that is intentionally light on required formatting or semantic standards. This is to encourage and facilitate rapid exploration and innovation. As individual components gain traction with the community, external IDs can be used to map the payload ID to external existing health standards using a uniform resource name (URN) to the BioPortal server [21], an approach that is similar to the Substitutable Medical Apps, Reusable Technologies (SMART) platform [22] (eg, the URN of Logical Observation Identifiers Names and Codes [LOINC] code [23] for serum sodium values would be purl.bioontology.org/ontology/LNC/2951-2). Developers may choose to map to zero or more external standards, and all component IDs will be indexed for search functions that will be available in the Open mHealth code repository.

### An Illustrative Use Case

An excellent use case that demonstrates the power of our architectural approach is self-care tools for posttraumatic stress disorder (PTSD). PTSD Coach is a mobile app conceived by the US Department of Veterans Affairs and Department of Defense to help PTSD patients manage acute distress symptoms through education, connection with personal and public resources, self-assessment, and personalized, interactive tools rooted in cognitive behavioral principles [24].

Management of PTSD presents an ideal use case for augmentation with mHealth tools, as many patients who need care may not seek in-person assistance due to stigma, logistical barriers, or lack of problem recognition [25]. While standard face-to-face treatments for PTSD have been found to be quite effective, mobile apps can provide a convenient, location-independent, anonymous alternative to standard care. Even those individuals who are receiving PTSD care may experience distress in the week that passes between treatment sessions. For them, mHealth can provide just-in-time tools, including crisis management strategies, wherever and whenever they are needed. Primary reliance on mobile devices for Internet access is becoming increasingly common [26], but existing PTSD Web resources are not optimized for mobile use.

Whether used between clinic visits or for independent self-management, PTSD Coach supports skill acquisition for coping with acute symptoms (eg, guided relaxation or progressive muscle relaxation); self-assessment for improved problem recognition and self-monitoring; and education aimed at increasing knowledge about PTSD and its effective treatments, decreasing stigma, providing messages of normalization, and increasing likelihood of entering care. Links to support, both national and personal, improve the individual’s chances of entering care if it is warranted. Due to data sensitivity concerns, PTSD Coach was built as a stand-alone application for patient self-care with no transmission of data to or from the app for clinician involvement. Open mHealth collaborated with the PTSD Coach team to develop a version called PTSD Explorer that captures and reports user-reported and other data back to a HIPAA-compliant server. PTSD Explorer will be integrated into the Veterans Administration’s electronic health record in a future phase of this project. Open mHealth’s approach to electronic and personal health record integration is not yet defined but will follow principles aligned with the “substitutable apps” approach described by Mandl and Kohane [27].

To help clinicians make sense of PTSD Explorer patient data for use in direct clinical care, we developed InfoVis data processing and visualization modules, some of which are generically usable across disease conditions. Data input and output formats of each InfoVis unit are specified as part of the DPU or DVU interface. Open mHealth’s modular open approach facilitated rapid exploration and iterative innovation to support participatory design of PTSD Explorer with a team of clinical psychologists and psychiatrists, allowing for quick and easy configuration of new dataviews for various clinicians, cohorts, and conditions and over time.

Our development process for PTSD DPUs and DVUs explicitly involved abstracting common processing functions that would be reusable across multiple disease conditions. For example, one DVU we built for PTSD Explorer displays continuous data over time, which is a disease-independent function that can be chained with other components to yield more complex, disease-specific visualizations (eg, of PTSD Checklist scores or blood glucose values over time). We are now using the DPUs and DVUs built for the PTSD use case to generate patient-facing visualizations of various self-reported measures of chronic pain. Because the Open mHealth community will reuse and adapt these DPUs, DVUs, and their interfaces over time, multiple approaches to processing and visualizing PTSD and pain data will coexist and will be reused or not depending on their effectiveness and value for both disease-independent and disease-specific usage.

Over time, actual usage and demonstrated value of InfoVis components across the range of all Open mHealth projects will drive convergence on common interface and semantic usage standards. DPUs and DVUs will process data from data storage units that access a wide range of third-party data applications and stores. In this modular way, Open mHealth will build a strong, community-sourced open component architecture to complement proprietary innovations to maximize the overall impact of mHealth.

## Personal Evidence Architecture

To further make sense of mHealth data streams, we have also designed a personal evidence architecture based on (1) standardized, validated clinical measures, 2) ways of collecting and interpreting these measures over time (such as statistical and graphical methods for time series analysis), and (3) use of an n-of-1 trial structure to reduce bias.

### Standardization of Clinical Measures in mHealth

To aggregate data collected across multiple mHealth applications and n-of-1 studies, we must first adopt a standardized clinical vocabulary. As the basis for our personal evidence architecture, we are incorporating measures from a Patient Reported Outcomes Measurement Information System (PROMIS) put forth by the National Institutes of Health [28]. These PROMIS measures are a system of patient-reported health outcome assessments for physical, mental, and social well-being. These measures are broadly validated, having been widely used as primary or secondary end points in clinical studies of treatment effectiveness across disease conditions.

In addition to traditional clinical measures, there remains a need to develop and validate measures specifically for mHealth, which enables self-reported data to be collected several times a day, rather than once every few months. One of the greatest benefits of mHealth will be using passively collected data to estimate and predict health outcomes, and we anticipate a flurry of activity around the design and validation of these approaches. We are exploring a shared set of metadata tags to capture the contextual variables about how data are collected to ensure that data and evidence can make sense together, as well as separately.

Standardized measures and vocabulary specific to mHealth would enable the aggregation of high-value mHealth data, greatly expanding its potential to advance the public good. Data access is a priority of the US government’s Open Government Initiative, as exemplified by the flagship Community Health Data Initiative at the US Department of Health and Human Services [29]. Combining mHealth data with other community health data streams, such as the US Department of Veterans Affairs’ Blue Button personal health data initiative, would catalyze the information ecosystem, expanding the potential use and applicability of mHealth data in guiding clinical decision-making, performance improvement, and community public health initiatives.

### N-of-1 Study Design

An n-of-1 study is a single-patient crossover trial in which an individual patient is randomly assigned to alternative interventions over time [30]. N-of-1 trials are most readily applicable to conditions that are chronic and to treatments that have a short onset and rapid washout. In contrast to anecdotal observations, n-of-1 trials can be used to identify effective approaches for an individual patient with enhanced scientific rigor [30-32].

Hundreds of n-of-1 studies have been completed and have been shown to be a rigorous means by which to generate personalized evidence [33]. However, n-of-1 trials have not gained much traction with clinicians, patients, and the scientific community at large because of the perception that such trials demand too much time and effort from clinicians and patients [34,35]. Mobile devices are well suited to overcome these barriers, as they facilitate data collection with minimal effort required by the physician and friction by the user.

Using our Personal Evidence Architecture, patients and clinicians (either together or independently) will be able to define a question, set up a study using an n-of-1 study template, and run the study on any mHealth app using our data analysis modules. Textbox 1 shows an example of this. By engaging patients in their own care, n-of-1 studies can enhance shared decision making, support better patient–clinician communication, and foster commitment to treatment, leading to better adherence. Furthermore, the results of n-of-1 trials can be aggregated using Bayesian methods, informing care of populations beyond the n-of-1 trial participants themselves. This would flip the traditional direction of research inference on its head, aggregating individual-level evidence to get at population-level evidence, rather than the other way around.

**Jack’s N-of-1 Trial.**

Jack is a 55-year-old man with chronic back pain of moderate severity. He is taking Vicodin 5/500 tablets several times daily, but the pills make him sleepy and he’s not sure they do much. Jack has been followed closely by Nurse Practitioner (NP) Erlich for several years. He is randomly assigned to the Trialist and decides to design an n-of-1 trial comparing Vicodin 5/500 5 tabs daily with acetaminophen 500 mg 5 tabs daily. Working with NP Erlich, he decides on 1-week treatment periods for a total of 6 weeks. In addition to “pain interference” (a mandatory outcome), he creates “longest time, in minutes, able to sit continuously at work” as his customized outcome.

These choices are programmed into the Trialist. Beginning the next day, Jack is notified to start acetaminophen. He is also reminded at random intervals to note how long he has been sitting and to what extent he is experiencing discomfort. Once weekly he reports on mandatory outcomes. The process continues for 6 weeks, with the Trialist signaling Jack to switch at intervals. Based on Jack’s n-of-1 trial results plus priors supplied at the beginning of the study, the Trialist reports that there is a <30% chance that Vicodin is superior to acetaminophen with respect to prolonged sitting and only a 10% chance for reduced pain interference. Jack decides to go with plain Tylenol. He does well, and 6 months later he tells Ms Erlich he received a promotion at work.

## Building the Open mHealth Community

Open mHealth is leading several ongoing projects, including work with the US federal government on PTSD and chronic pain as discussed above, to demonstrate the efficacy of an open architecture and the value of a broad, open community. For each project, we consider and aim to support all three feedback loops of self-care, clinical decision making, and research evidence to maximize clinical and scientific impact. These projects exemplify the value of joint technical and health innovation, since the high-level features are determined by what is clinically relevant (eg, mobility correlates of chronic pain), while the lower-level features are determined by what is technically feasible (accelerometer data from onboard phone sensors).

Open mHealth, and by extension mHealth, would be more successful with more projects where health innovators and developers can jointly develop tools and methods that are then shared through an open architecture. To catalyze this community, we are (1) convening capacity-building workshops, to increase the number of health innovators using Open mHealth, (2) holding developer connection events to galvanize the developer community, and (3) creating self-governing working groups to advance work in key topic areas.

Our paramount community engagement goal is to make it as easy and as worthwhile as possible for health and technology innovators to use and contribute to Open mHealth and to advance overall mHealth impact and effectiveness. While we do not expect all of mHealth to be open, we hope to foster a commons for sharing and learning that is inclusive of proprietary components and approaches, to allow health innovators and entrepreneurs to focus on their unique market offerings while increasing the validity, robustness, and efficiency of shared components and methods. In addition, adoption of our open architecture and Personal Evidence Architecture components will help generate more evidence that can be pooled and shared across studies, resulting in a stronger, more cohesive evidence base on mHealth efficacy for personalized care.

Open mHealth is the only organization dedicated to scaling effective mHealth solutions through an open architecture and an open collaborative community drawn from both the health and technology realms. Open mHealth is different from other organizations such as AT&T and Aetna that are developing open end-to-end platforms for mHealth applications, in that Open mHealth modules are embeddable within applications developed on those platforms. Open mHealth is not a competing platform but a source of shared components that can be compatible with AT&T and other open and closed platforms. Open mHealth is also different from organizations such as the mHealth Alliance or the nascent National Institutes of Health-led mHealth Public–Private Partnership, which are dedicated to scaling mHealth through public–private partnerships but not through a technical infrastructure. Open mHealth’s overall approach and the specific software developed for InfoVis and Personal Evidence Architecture are applicable within those partnerships, and Open mHealth continues to be active in collaborating with the myriad other organizations in the mHealth space.

### Conclusion

While mHealth holds great promise, disappointment in health information technology has been commonplace, with hype cycles that come and go, punctuated by successful but ultimately limited pilots. At this juncture, improving health outcomes faster and at lower cost is essential. We look to adapt the model of one of the most successful innovations of all time—the Internet—to Open mHealth to seed and catalyze methods and techniques for maximal improvement of individual and population health through a vibrant and open mHealth community.

## Abbreviations

API: application programming interface
DPU: data processing unit
DVU: data visualization unit
HIPAA: Health Insurance Portability and Accountability Act
LOINC: Logical Observation Identifiers Names and Codes
PROMIS: Patient Reported Outcomes Measurement Information System
PTSD: posttraumatic stress disorder
SMART: Substitutable Medical Apps, Reusable Technologies
